# Teaching Macrosystems Ecology Concepts With a Collaborative, Adaptable Education Module

**DOI:** 10.1002/ece3.73909

**Published:** 2026-07-01

**Authors:** Megan C. Malish, Shang Gao, Daniel C. Allen, Thomas M. Neeson

**Affiliations:** ^1^ Department of Geography and Environmental Sustainability University of Oklahoma Norman Oklahoma USA; ^2^ School of Natural Resources and the Environment University of Arizona Tucson Arizona USA; ^3^ Department of Ecosystem Science and Management Pennsylvania State University State College Pennsylvania USA

**Keywords:** collaborative learning, cross‐scale interaction, ecology education, macrosystems ecology

## Abstract

Macrosystems ecology offers a powerful set of concepts and tools for understanding large‐scale environmental challenges. Training students to understand and apply these ideas can be challenging, as it requires students to engage with complex concepts and advanced technical skills. To address these challenges, we developed an education module to teach students the conceptual and technical skills necessary for macrosystems ecology research. The relatively short, two‐part module facilitates students' exploration of the role of cross‐scale interactions, a fundamental macrosystems concept, through applications to stream hydrology. We implemented the education module in a university course with mixed undergraduate and graduate student enrollment and used learning assessments to evaluate the effectiveness of the module. We found that the module increased students' self‐reported proficiency and confidence in understanding and interpreting large geospatial datasets. Students also demonstrated increased knowledge of cross‐scale interactions after completion of the module. Notably, we found evidence that synthesis of ideas through collaborative learning was a key driver of gains in student outcomes. Our study demonstrates an effective approach for teaching difficult concepts and skills relevant to macrosystems ecology research. Our education module is flexible and adaptable, and could be implemented broadly in STEM courses to teach macrosystems concepts to a wide range of students.

## Graphical abstract

We designed an education module to teach students the conceptual and technical skills necessary for macrosystems ecology research. The adaptable and flexible module improved student proficiency and confidence in working with large, geospatial datasets and student knowledge of cross‐scale interactions.

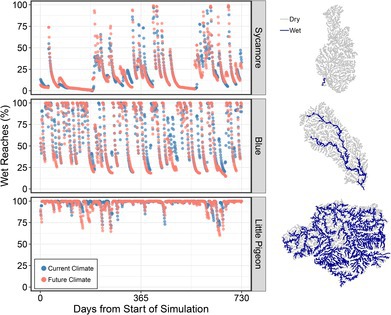

## 1 Introduction

Many grand environmental challenges are global in scope (Reid et al. 2010). Addressing these challenges requires an understanding of ecological patterns and processes at broad spatiotemporal scales, their interactions with one another, and with patterns and processes at other scales. These concepts and the methods used to study them are commonly labeled *macrosystems ecology* (Heffernan et al. 2014). In recent years, ecological research has increasingly shifted toward macrosystems ecology and related fields of study (Fei et al. 2016; McCallen et al. 2019). This growth is attributed in part to increased attention toward major global challenges, such as climate change and land‐use change, that require large‐scale, transdisciplinary approaches (Dodds et al. 2021). Additionally, methodological and technological advancements have contributed to the proliferation of macrosystems ecology research. Advances in data collection methods, including remote sensing and distributed data collection networks, have expanded the availability of large‐scale data sets required for macrosystems ecology research (McCallen et al. 2019). At the same time, the computational and statistical tools needed for analysis of these datasets have become readily accessible to ecologists (Heffernan et al. 2014; Levy et al. 2014; McCallen et al. 2019; Zipkin et al. 2021).

To understand and apply macrosystems ecology concepts, students must be trained in multiple skillsets. First, students need systems thinking to understand and address complex challenges (Hogan and Weathers 2003; Weathers et al. 2016; Carey et al. 2020). This includes learning how interactions between individual components of a system affect the overall system, enabling students to engage with key macrosystems concepts such as cross‐scale interactions (Heffernan et al. 2014). Second, students should be taught the technical skills needed to work with large datasets. These skills include data management, integration of datasets, and advanced statistical techniques for analysis (Michener and Jones 2012; Durden et al. 2017). Finally, interpersonal skills are often necessary to carry out macrosystems research. Because macrosystems research occurs over large areas and often across disciplines, it frequently requires communication and collaboration among scientists (Cheruvelil et al. 2014; Goring et al. 2014; Farrell et al. 2021). Together, these conceptual, technical, and interpersonal skills highlight that successful preparation of students requires teaching a diversity of skillsets.

Despite the recognized value of preparing students to think and work at macroscales, many challenges can prevent relevant skills from being learned in the classroom. These challenges can include difficulty teaching and poor student comprehension of fundamental concepts, such as scale (Cheek et al. 2017), and more complex derivatives, such as cross‐scale interactions. Students may also be intimidated by learning curves associated with technical skills necessary for macrosystems research (Cartile 2020). In many undergraduate and graduate classrooms, interpersonal skills may not be taught or practiced at all. This lack of training in key skills persists at advanced career stages. Among macrosystems researchers, a mismatch has been identified between the skills perceived as important for macrosystems research and the training that they have received (Farrell et al. 2021). Thus, formalized training in conceptual, technical, and interpersonal skills in the classroom can benefit macrosystems researchers at all career stages.

To address the challenges associated with teaching macrosystems ecology in the classroom, we developed an education module to teach students research approaches and skills that are important for macrosystems ecology research. The module facilitates students' exploration of the role of cross‐scale interactions, a fundamental macrosystems concept, through applications to stream hydrology. Students also practice technical and interpersonal skills throughout the module. To overcome the challenges of conceptual complexity and intimidation, we designed the module using a scaffolding approach, in which students are guided through a series of increasingly conceptually and technically complex steps. We implemented the module in a mixed‐enrollment (undergraduate and graduate) *Spatial Statistics* course and used learning assessments to evaluate the effectiveness of the module. Specifically, we examined how the module affected (1) students' self‐reported proficiency and confidence in working with large‐scale hydrological datasets and (2) students' perceptions and knowledge of cross‐scale interactions.

## 2 Methods

### 2.1 Module Overview

We designed an education module that enabled students to explore hydrological modeling on their own and then, working in small groups, discover how cross‐scale interactions between regional and global processes affect their focal stream networks. Specifically, the learning objectives of the module are (1) to understand how climate change influences hydrology in a specific stream network, (2) understand how cross‐scale interactions between global climate change and regional processes modulate the effects of climate change on stream network hydrology, and (3) understand how macrosystems scientists use models and code to quantify spatial and temporal patterns in hydrology (Table 1). Students learn how a global scale process—climate change—interacts with regional processes (e.g., geology, landcover, and topography) to drive changes in stream drying patterns in stream networks.

**TABLE 1:** The three learning objectives of the education module and associated lab components.

Learning objective	Associated lab component
Obj. 1: Understand influences of climate change on hydrology in a specific stream network	Part A: Understanding Stream Drying Patterns
Obj. 2: Understand how cross‐scale interactions between global climate change and regional processes modulate the effects of climate change on stream network hydrology (i.e., Obj. 1)	Part B: Comparing Stream Drying Patterns
Obj. 3: Understand how macrosystems scientists use models and code to quantify spatial and temporal patterns in hydrology	Parts A and B

Our two‐part module was designed to lead students to discover key macrosystems concepts through a combination of individual and group work (Table 1). The education module materials consist of the data needed to complete the module and an instructional document, provided as a pdf, that guides students through the module. The document contains R code, R outputs, and accompanying text explanations of code and output. The module assumes no prior knowledge of R or hydrologic modeling. Further, the module was specifically developed to introduce students to realistic hydrologic data analyses. For example, instead of using simpler point and click type exercises, students are asked to modify provided code to complete portions of the education module. Additionally, prompts and questions are included throughout the instructional document to encourage students to critically engage with their findings. While the module focuses on stream hydrology, it was designed to be broadly applicable to earth science and ecology courses. Therefore, the instructional document also includes a background section that introduces key macrosystems ecology concepts. Instructors may choose to supplement this introduction with additional information relevant to their course.

**FIGURE 1:**
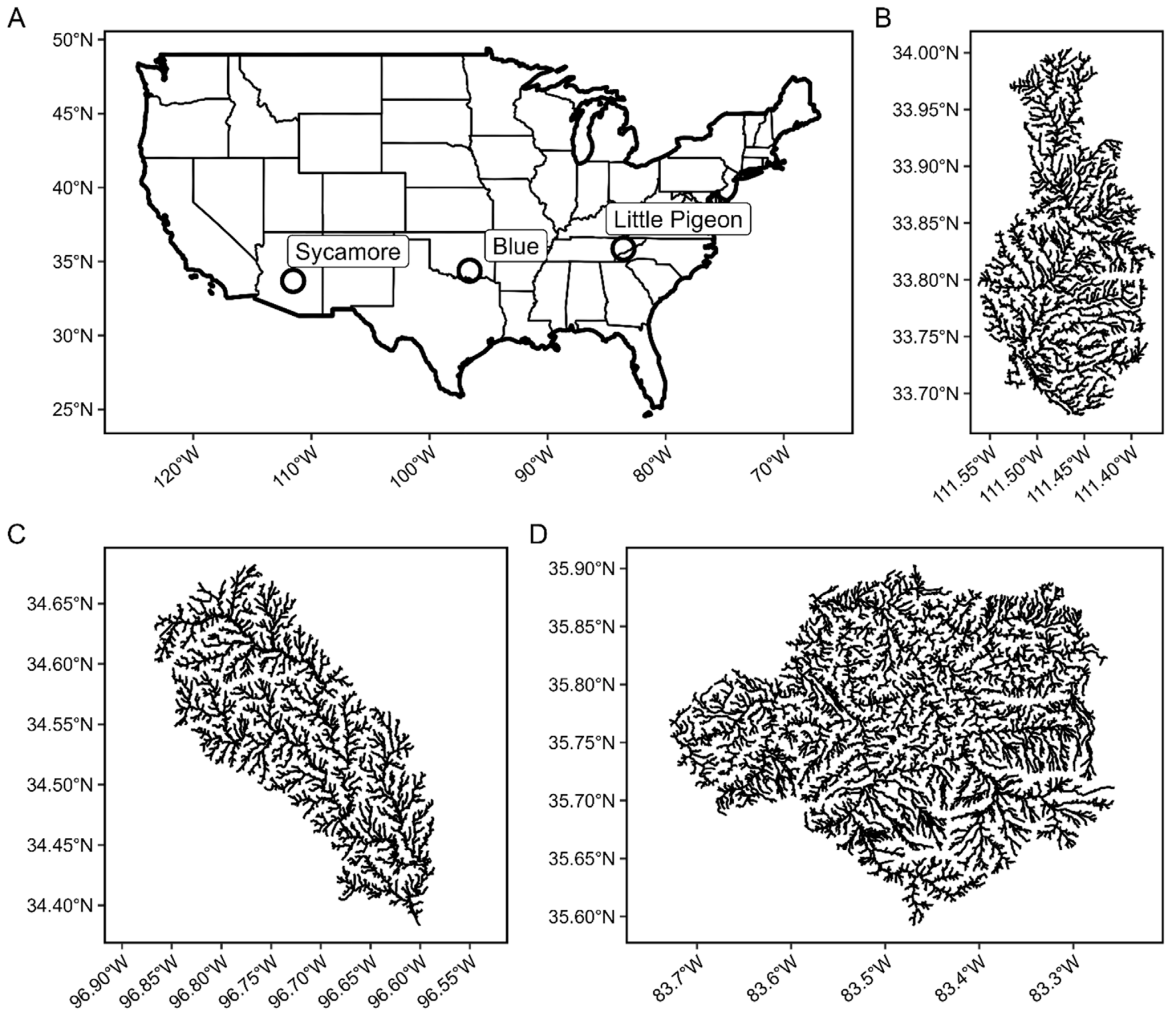
Locations (A) and maps of the study stream networks: (B) Sycamore Creek, AZ; (C) Blue River, OK; (D) Little Pigeon River, TN.

The first part of the module, *Part A: Understanding Stream Drying Patterns*, is completed individually by students and is estimated to take 90 min. There are three versions of Part A, each focused on a different stream network in the United States (Figure 1). Each version of Part A provides contextual information about the focal stream network, such as mean annual temperature and precipitation. Students receive two types of data to complete Part A. They are given a spatial dataset of the focal stream network and two tabular datasets with daily streamflow values. Both streamflow datasets contain 2 years of modeled daily streamflow data for each stream reach in the stream network, and are subsets of hydrological model outputs previously published (Malish et al. 2025). One streamflow dataset represents a current climate scenario. It was modeled using a climate dataset based on observed data (Dee et al. 2011). The other streamflow dataset represents a projected future climate. The climate data used to create this output was created by adding a climate perturbation to the current climate dataset to simulate climate change. The climate perturbation is equal to the 95‐year CMIP5 multi‐model ensemble mean change signal under the RCP8.5 emission scenario, which corresponds to an approximately 3°C–6°C warming signal (Liu et al. 2017). The spatial and tabular datasets are linked with a common identifier.

Part A is divided into three sections: *prepare data*, *visualize stream drying*, and *measure stream drying*. In the *prepare data* section, students are guided through preparing their R environment by installing the necessary packages and loading the provided data. The *visualize stream drying* section familiarizes students with the datasets through visualizations and summary statistics. Finally, students use a set of quantitative metrics to measure stream drying and its changes through time and between climate scenarios in the *measure stream drying* section. The final prompt in Part A asks students to spend 5–10 min researching local and regional processes that may influence stream drying patterns.

**FIGURE 2:**
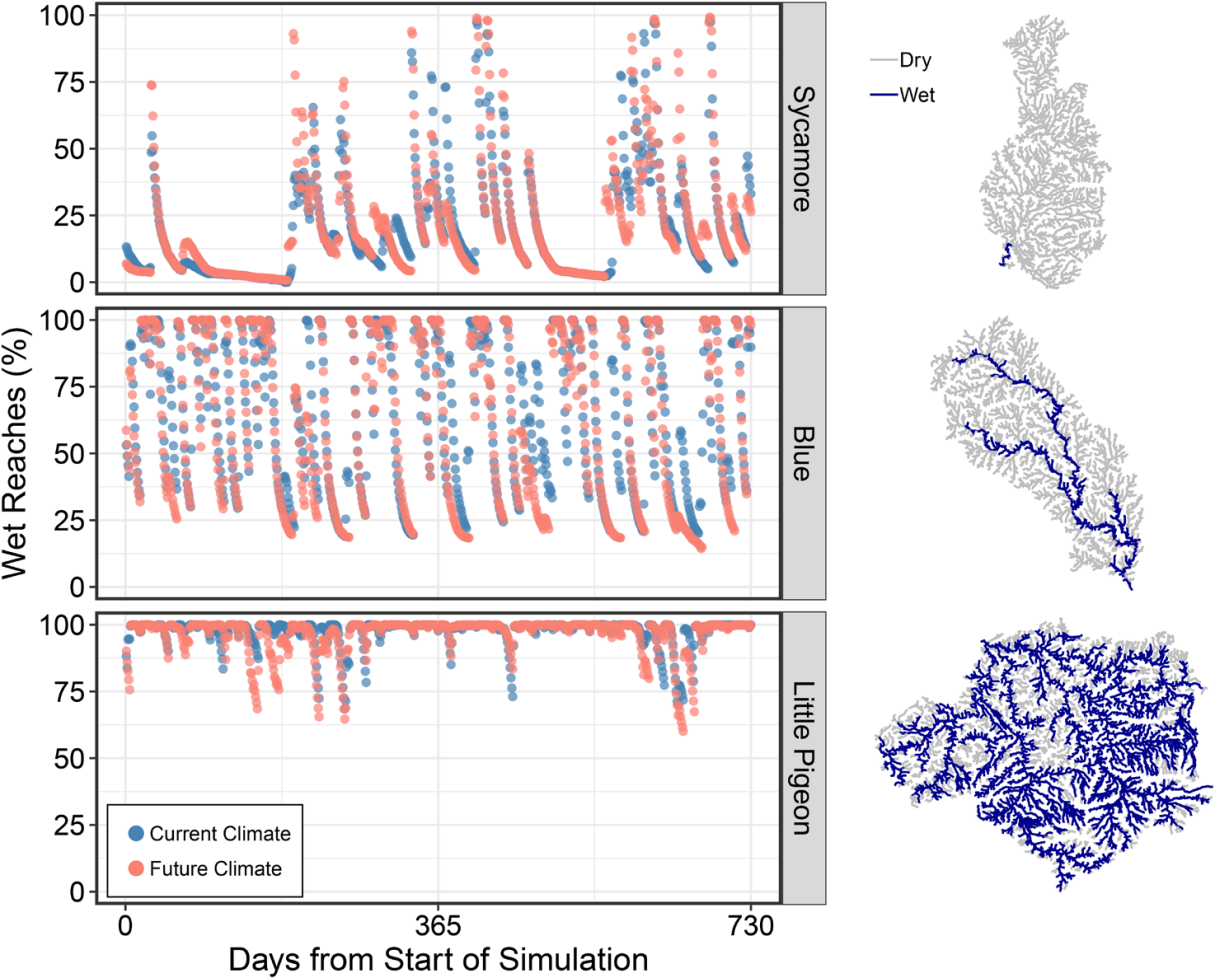
Students observe the temporal and spatial drying patterns of drying in the three study watersheds by plotting time series and creating maps. (A) The timing, frequency, and extent of drying vary among the study watersheds. (B) The spatial drying patterns also vary among the watersheds. Maps show the greatest drying extent from the current climate scenario.

The second part of the education module, *Part B: Comparing Stream Drying Patterns*, emphasizes the synthesis of findings through student collaboration. Part B is completed by students in groups and is estimated to take 30 min. Groups are composed of at least one student who focused on each stream network in Part A, resulting in a minimum of three students per group. Here, each student acts as an expert on their focal stream network. To complete Part B, each student shares their findings from Part A and students together synthesize findings through the discussion of similarities and differences in spatial and temporal drying patterns among stream networks (Figure 2). Next, students cooperate to create new visualizations to directly compare stream drying patterns among the stream networks. Finally, students work together to answer a series of questions that help them to draw conclusions about how cross‐scale interactions influence stream drying patterns. Groups receive a dataset of the drying metrics that were calculated in Part A for all three stream networks, ensuring that difficulties encountered in Part A do not limit participation in Part B. A key idea that students learn from the module is that the impacts of climate change on future stream drying patterns differ among the stream networks. The hydrologic analyses done by students show that two stream networks in the module experience more stream drying in the future, while stream drying is expected to decrease in the third. In other words, the effects of climate change, a global process, are mediated by regional processes resulting in different impacts on stream hydrology.

The education module was designed to be flexible and adaptable. Module materials are publicly available on QUBES (Malish et al. 2026). In addition to the materials described above, editable R Markdown files that were used to create the instructional documents are also available. Instructors can edit the files to alter the module to best suit their course. For example, instructors could edit the exercises or follow‐up questions to align with course learning outcomes. Additionally, instructors might reduce the amount of code provided to challenge students to develop their own code. Finally, instructors may wish to include additional stream networks. Hydrologic model outputs and shapefiles are available for eight additional stream networks from across the United States (Malish et al. 2025). With this flexibility, the education module can be successfully implemented in a variety of courses.

### 2.2 Implementation and Assessment

We implemented the module in a mixed‐enrollment (undergraduate and graduate students) Geographic Information Systems (GIS) course on spatial statistics at the University of Oklahoma. The course is required for several degree programs including GIS B.S., Geospatial Technologies M.S., and Environmental Systems M.S., resulting in a diverse enrollment of students with wide‐ranging experience and comfort with statistics, coding, and spatial analysis. The course also attracts students from a variety of other degree programs, including those pursuing degrees in biology and civil engineering. A total of 26 students participated in the study. We did not collect data on student education level or educational background because we were interested in understanding the effectiveness of the module at teaching conceptual and technical skills required for macrosystems research for a broad array of students.

We evaluated the effectiveness of the education module with a series of online assessments. Assessments were taken by students immediately before starting Part A, immediately following Part A, and immediately following Part B. We considered these the pre‐, mid‐, and post‐stages, respectively. Links to the online surveys were provided within the instructional document to encourage students to complete the surveys. Assessments at each stage were identical and were administered online with no time limits or restrictions on materials that could be used to answer the questions. Student responses were recorded anonymously. Only student participants who voluntarily consented to the use of their data were included in our study following our Institutional Review Board protocol (University of Oklahoma IRB #16234).

The assessments contained three groups of questions to assess student growth (Table 2; Appendix S1). First, students were asked to rank their perceived proficiency and confidence in understanding and interpreting hydrological data. Students were also asked to rank their knowledge of cross‐scale interactions. These self‐assessments were done using a Likert‐type scale from low (1) to high (5). A second set of questions focused on students' ability to define, interpret, and apply macrosystems concepts. These three processes (*define*, *interpret*, *apply*) represent scaffolded components for high level learning (Anderson and Krathwohl 2001; Fink 2013; Hounshell et al. 2021). Students completed a free‐response question where they were asked to *define* cross‐scale interactions in the context of stream hydrology. Finally, a series of three multiple choice questions evaluated students' ability to *interpret* data visualizations similar to those they encountered in the module and to *apply* concepts by making predictions about stream drying patterns in familiar and new contexts. We expected gains in technical and conceptual skills to differ among stages. We expected gains related to technical skills (Questions 1, 2, 5, and 7; Table 2) to occur after Part A, because most of the data processing, visualization, and analysis occurs at this stage. In contrast, we expected the largest gains related to conceptual understanding of cross‐scale interactions (Questions 3, 4, and 6; Table 2) to occur after Part B, because of the synthesis that occurs at this stage.

**TABLE 2:** Assessment questions.

	Question type	Question	Response type
1	Self‐assessment	How would you rank your proficiency with understanding and interpreting hydrological data?	Likert‐type scale
2	Self‐assessment	How would you rank your confidence with understanding and interpreting hydrological data?	Likert‐type scale
3	Self‐assessment	What statement best describes your current knowledge of cross‐scale interactions?	Likert‐type scale
4	Define	To the best of your ability, describe what “cross‐scale interactions” means in a hydrological context.	Free response
5	Interpret	The following figure shows 2 years of data for a watershed. In the figure, what is a possible cause of the reduced percent wet length that occurred between May and September?	Multiple choice
6	Apply	You are modeling streamflow in a watershed with non‐perennial reaches for a 5‐year period. In the first simulation, you run the model to simulate stream flow for current climate conditions. In the second simulation, you run the model to simulate stream flow for climate conditions expected 50 years from now. How will stream drying, as measured by percent wet length, differ between the two simulations?	Multiple choice
7	Apply	You model stream flow in a watershed for current and future climate conditions and find that stream drying increases under future climate conditions. How would you expect percent wet length and number of dry segments to differ between current and future stream flow simulations?	Multiple choice

### 2.3 Assessment Analysis

We analyzed assessment responses using standard methods (Miles and Huberman 1994). We first tested for changes in student self‐perception of proficiency, confidence, and knowledge among the three stages of the module. To test for differences in student perception of proficiency and confidence in working with hydrologic data, we used Kruskal–Wallis rank sum test to compare the mean Likert scores among groups and, when a difference was present, we used pairwise Wilcoxon tests with a Bonferroni correction for post hoc pairwise comparisons. We followed the same approach to test for changes in students' self‐reported knowledge of cross‐scale interactions. To analyze the multiple‐choice responses, we first tabulated the number of students who answered each multiple‐choice question correctly and incorrectly. We then used a chi‐squared test to identify differences in the frequencies of correct answers among stages.

To assess student responses for the free response question, we used a standardized coding method (Miles and Huberman 1994). Responses were coded in two phases. During the first step, we preliminarily reviewed student responses and recorded emerging themes and keywords. We then developed a codebook with four emerging themes associated with correct responses and keywords associated with each theme. During codebook development, all information regarding survey timing (pre‐, mid‐, or post‐stage) was hidden and responses were randomly ordered using a random number generator. At the second step, we identified the themes mentioned in each student response. We then tested for differences in the proportion of responses that included each theme using chi‐squared tests. When differences among stages were identified, we conducted post hoc pairwise comparisons with a Bonferroni correction.

All analyses were conducted in R (v.4.4.1; R Core Team 2024).

## 3 Results

### 3.1 Student Self‐Assessment of Proficiency, Confidence, and Knowledge

**FIGURE 3:**
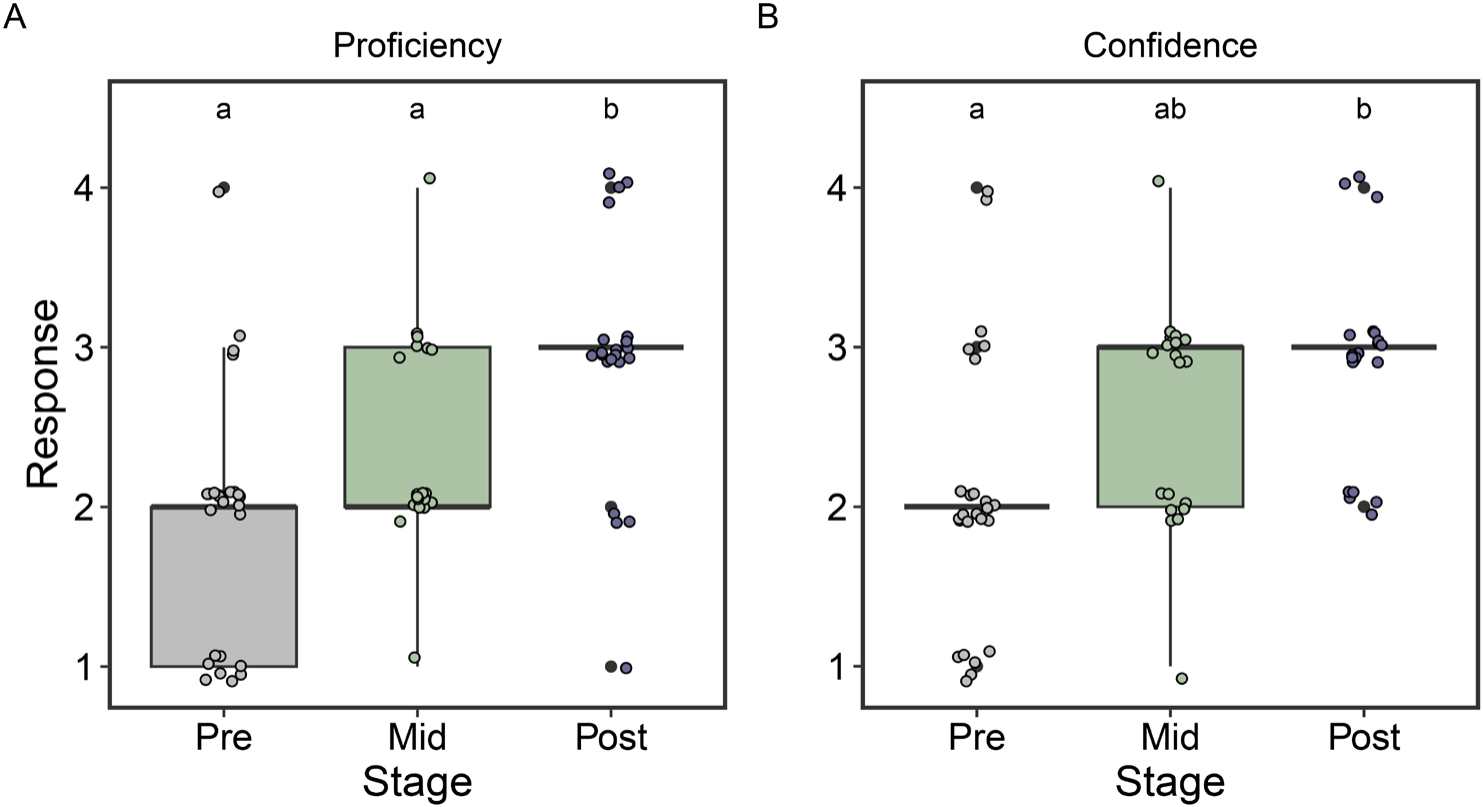
When asked about working with hydrologic data, students reported (A) greater proficiency (Question 1) at the post‐stage than the pre‐ and mid‐stages and (B) greater confidence (Question 2) at the post‐stage than at the pre‐stage. Letters denote statistically significant pairwise differences (*p* < 0.05).

Completing the education module significantly increased students' self‐reported proficiency with and confidence in understanding and interpreting hydrological data (Figure 3, Table 3). On average, students' reported proficiency increased from “basic proficiency” to “intermediate proficiency” after completing the assessment. There was a significant difference in proficiency among stages (*χ* ^2^ = 19.912, *p* < 0.001). Post hoc analysis indicated that the proficiency at post‐stage (M = 3.0) was significantly greater than the pre‐ (M = 1.9, *p* < 0.001) and mid‐stages (M = 2.4, *p* < 0.05). Proficiency at the pre‐ and mid‐stages did not differ. Students' average confidence increased from “somewhat confident” before the module to “moderately confident” after the module. There was a significant difference in confidence among stages (*χ* ^2^ = 13.749, *p* = 0.001). Post hoc comparisons showed that confidence at the post‐stage (M = 2.9) was significantly different from the pre‐stage (M = 2.1, *p* < 0.01), but the mid‐stage (M = 2.6) did not differ from the pre‐ and post‐stages.

**TABLE 3:** Summary of scores and Kruskal–Wallis rank‐sum test results of student self‐reported proficiency, confidence, and knowledge at the pre‐ (*n* = 26), mid‐ (*n* = 20), and post‐stages (*n* = 21).

Metric	Pre‐stage score, M (SD)	Mid‐stage score, M (SD)	Post‐stage score, M (SD)	*χ* ^*2*^	*p*
Proficiency	1.9 (0.8)	2.4 (0.7)	3.0 (0.7)	19.912	< 0.001
Confidence	2.1 (0.8)	2.6 (0.7)	2.9 (0.6)	13.749	0.001
Knowledge	1.5 (0.7)	2.7 (0.6)	3.1 (0.7)	36.378	< 0.001

*Note:* Questions used a Likert‐type scale with responses ranging from 1 (low) to 5 (high).

**FIGURE 4:**
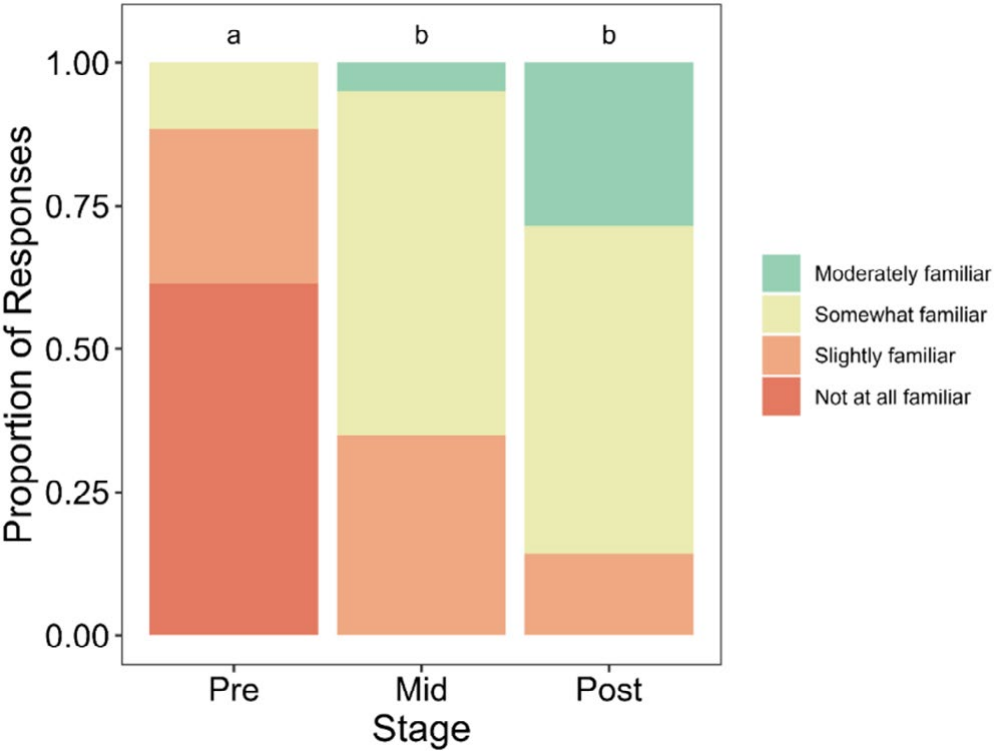
Students reported that their knowledge of cross‐scale interactions (Question 3) was greater at the mid‐ and post‐assessment stages than at the pre‐assessment stage. Letters denote statistically significant pairwise differences (*p* < 0.05). A fifth option, “Extremely familiar” was available but not chosen by any students.

Students' knowledge of cross‐scale interactions also significantly increased (*χ* ^2^ = 36.378, *p* < 0.001; Figure 4, Table 3). Most students reported that they were “not at all familiar” with cross‐scale interactions during the pre‐assessment. Following the education module, on average students reported they were “somewhat familiar”. Post hoc comparisons indicated that knowledge at mid‐ (M = 2.7) and post‐stages (M = 3.1) did not differ from each other, but both were significantly greater than the pre‐stage (M = 1.5, *p* < 0.001).

### 3.2 Student Ability to Define, Interpret and Apply Key Concepts

**FIGURE 5:**
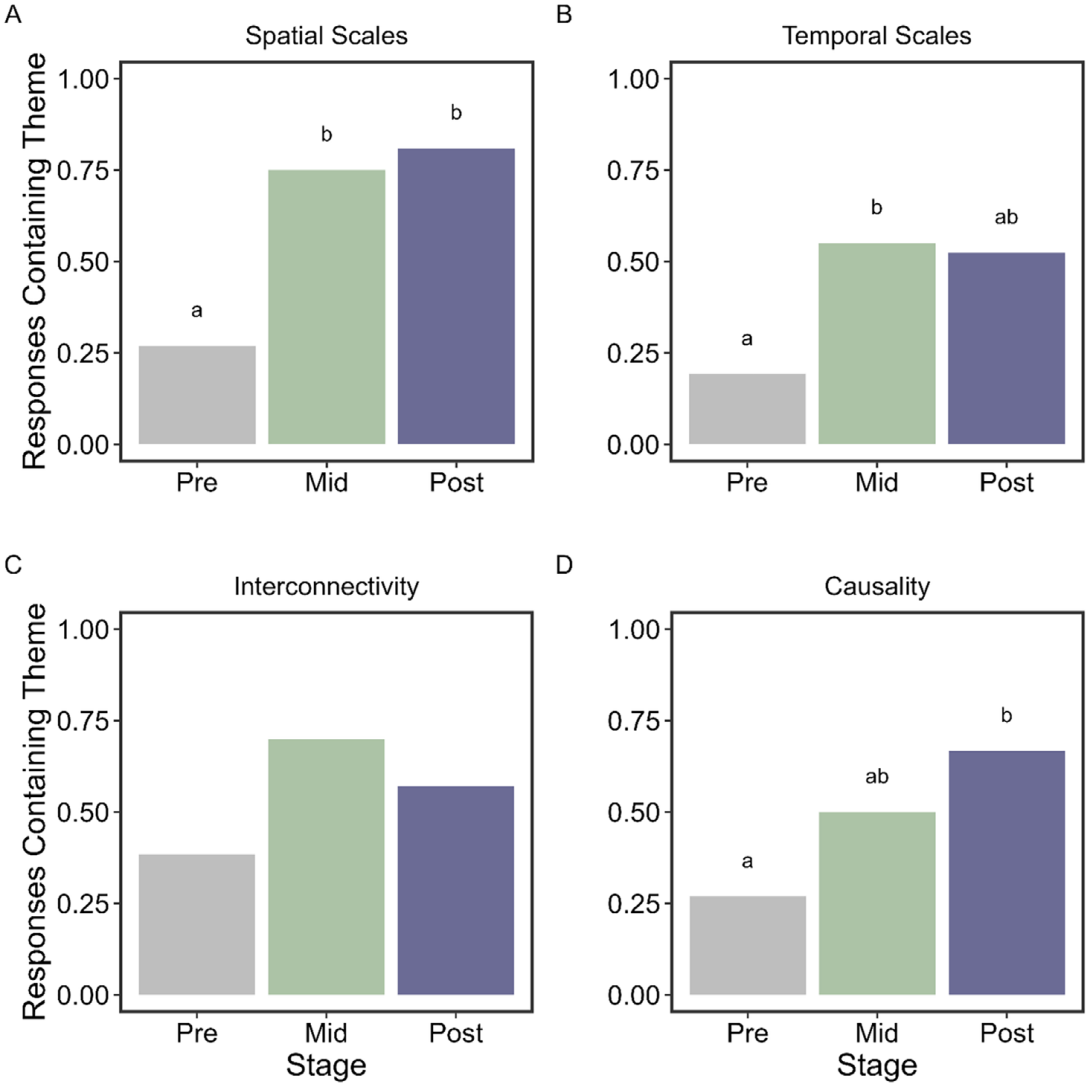
Proportion of the free response answers (Question 4) that included thematic keywords tended to increase at later stages of the module. Thematic keywords were (A) “spatial scales,” (B) “temporal scales,” (C) “interconnectivity” or interactions among scales, and (D) “causality” or processes at one scale having impacts at another. Letters denote statistically significant pairwise differences (*p* < 0.05).

In addition to evaluating students' self‐reported proficiency, knowledge, and confidence, we also examined students' ability to *define*, *interpret*, and *apply* key ideas regarding cross‐scale interactions at the three stages. Students were asked a free‐response question to define “cross‐scale interactions”. From student responses, we identified four emergent key themes associated with correct answers: *spatial scales*, *temporal scales*, *interconnectivity* among scales, and *causality* between such interactions and observable outcomes like stream drying. The frequency of mentions among stages differed for *spatial scales* (*χ* ^2^ = 17.25, *p* < 0.001), *temporal scales* (*χ* ^2^ = 7.87, *p* < 0.05), and *causality* (*χ* ^2^ = 7.54, *p* < 0.05), but not *interconnectivity* (Figure 5). Post hoc analyses showed that the frequency of mentions of *spatial scales* was significantly lower at the pre‐stage (freq = 0.27) than the mid‐ (freq = 0.75, *p* < 0.01) and post‐stages (freq = 0.81, *p* < 0.001), but no difference in frequency between the mid‐ and post‐stages. The frequency of mentions of *temporal scales* was significantly less at the pre‐stage (freq = 0.19) than the mid‐stage (freq = 0.55, *p* < 0.05), but the post‐stage (freq = 0.52) did not differ from the pre‐ and mid‐stages. The theme of *causality* was more frequently mentioned during the post‐stage (freq = 0.67) than the pre‐stage (freq = 0.27, *p* < 0.05), but the mid‐stage (freq = 0.50) did not differ from the pre‐ and post‐stages.

**FIGURE 6:**
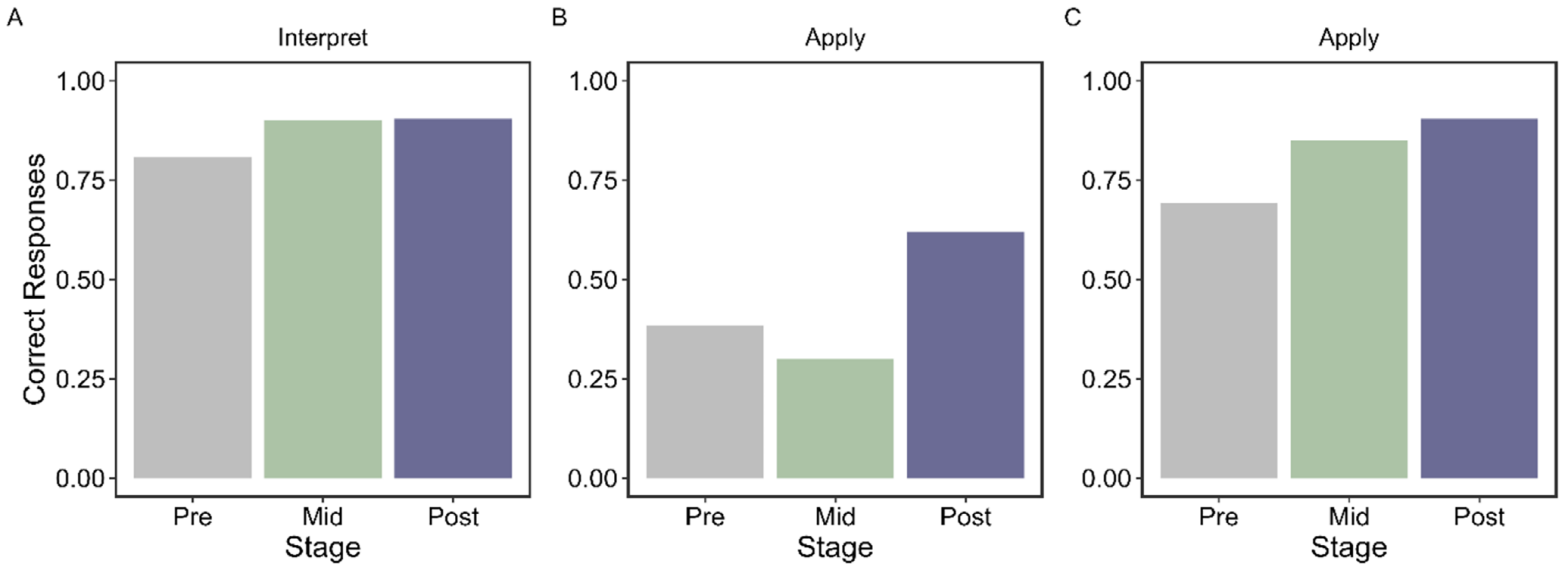
The correct response rate for multiple choice questions did not significantly differ among stages (*p* > 0.05). The multiple choice questions addressed students' ability to *interpret* in (A) Question 5, and *apply* in (B) Question 6 and (C) Question 7. Question 6 tested for conceptual understanding of cross‐scale interactions while Question 7 tested for technical understanding of quantitative metrics.

Three multiple choice questions assessed students' ability to *interpret* and *apply* key ideas. The frequency of correct responses ranged from 0.38 to 0.81 at the pre‐stage, 0.30 to 0.90 at the mid‐stage, and 0.62 to 0.90 at the post stage (Figure 6). The frequency of correct answers did not vary significantly among stages for any multiple‐choice question.

## 4 Discussion

We developed an education module that enables students to explore how cross‐scale interactions drive complex patterns in stream hydrology. Student assessments were overwhelmingly positive and revealed that our short, two‐hour module boosted their self‐confidence, knowledge, and perceived proficiency with challenging macrosystems concepts. Overall, our results demonstrate how integrated education modules can be an effective way to teach advanced concepts from macrosystems ecology to a range of students.

One channel through which student confidence and proficiency increased was by visualizing, analyzing, and discussing hydrologic model outputs. The confidence and proficiency gained in working with hydrological data are likely to be generalizable to other large datasets as well. While the observed increases in confidence and proficiency throughout the duration of the education module were moderate, they represent substantial benefits. For example, confidence is an important component of student success. Confidence in one's skills and abilities is an important aspect of student retention in STEM fields (Anderson et al. 2011). When their confidence is high, students are more likely to feel motivated to work toward academic goals, to persist during problem‐solving activities, and to stay enrolled in college (Pajares 1996; Gore 2006). Thus, the confidence that students gained through our learning module may boost their long‐term engagement with STEM and higher education, and provide a foundation for future growth.

Students also demonstrated increased knowledge of cross‐scale interactions after completion of the education module. We were unable to control for differences among students in our analysis, such as experience level or other demographic differences. Our study focused on comparing pre‐, mid‐, and post‐stage assessments of self‐confidence and proficiency among a single group of students, and reported changes are therefore representative of the students in the class. There is potential that changes in self‐confidence, knowledge, and proficiency would differ for other student populations. However, the gains we observed show that most students benefitted from the education module. Other studies have found similar success using hands‐on learning with large datasets to teach macrosystems concepts (Styers et al. 2021; Hounshell et al. 2021). The success of these modules across many universities, in a variety of course types (e.g., ecology, geography, GIS), and while using diverse data types (e.g., biodiversity, limnology, stream hydrology) is indicative of the utility of hands‐on education modules to introduce students to macrosystem concepts.

The synthesis of findings through collaborative learning among students was found to be a key driver of gains in students' perceived confidence and proficiency in working with hydrological data. We expected gains in conceptual skills, rather than technical skills, to be greatest following the synthesis portion of the module. While some gains in conceptual skills were identified at this stage, the gains related to technical skills were most notable. Indeed, statistically significant gains in student confidence and proficiency in working with hydrologic data occurred only after the collaborative component of the learning module. During this stage, students compared their individual findings and applied their knowledge to make new inferences. Synthesis and application requires higher level thinking than knowledge and comprehension alone (Anderson and Krathwohl 2001). Thus, to complete this portion of the module, students were required to critically engage with the information, which may have contributed to the observed gains in confidence and proficiency. Collaboration itself may have contributed to student gains, as well. Collaborative learning has been frequently shown to promote higher confidence in students than individual learning (Norem‐Hebeisen and Johnson 1981; Johnson et al. 2007). This seems to occur because group work allows students to internalize perceptions that they are accepted and to appreciate mutual success (Johnson and Johnson 1989). In addition to increasing confidence, collaborative learning has many other benefits. Compared to individualistic learning, collaboration leads to greater student achievement, knowledge retention, and use of higher‐level reasoning (Tinto 1993; Johnson et al. 2007; Yamarik 2007). Collaborative learning also teaches students to be good collaborators. Students learn to communicate their ideas and constructively resolve conflicts (Mendo‐Lázaro et al. 2018; Loh and Ang 2020). The development of communication skills is especially pertinent to macrosystems ecology, a field of study that frequently requires collaboration among scientists with varying expertise (Cheruvelil et al. 2014; Farrell et al. 2021). Educational modules such as ours that develop conceptual, technical, and interpersonal skillsets contribute to the preparation of the next generation of macrosystems researchers.

## 5 Conclusion

Our study demonstrates an approach for students to explore, discuss, and discover difficult, abstract concepts from macrosystems ecology. While macrosystems concepts can be complex, successfully teaching students these concepts can be made possible through a flexible and adaptable hands‐on education module. The module we presented is short (approximately 2 h), yet measurably increased student confidence, proficiency, and knowledge. The structure of the module includes designed higher order thinking facilitated through collaboration and discussion among students, which drove gains in student confidence and proficiency. Given that our lab module was implemented in a course in which students had a variety of disciplinary backgrounds, many with limited exposure to ecology, our module could be implemented broadly in general STEM courses to teach macrosystems concepts to a wide range of students.

## Author Contributions

**Megan C. Malish:** conceptualization (equal), formal analysis (equal), investigation (equal), visualization (equal), writing – original draft (equal). **Shang Gao:** investigation (equal), writing – review and editing (equal). **Daniel C. Allen:** funding acquisition (equal), investigation (equal), project administration (equal), supervision (equal), writing – review and editing (equal). **Thomas M. Neeson:** conceptualization (equal), funding acquisition (equal), investigation (equal), project administration (equal), supervision (equal), writing – review and editing (equal).

## Funding

This work was supported by Directorate for Biological Sciences (DEB‐1802766, DEB‐2207680).

## Conflicts of Interest

The authors declare no conflicts of interest.

## Supporting information


**Appendix S1:** ece373909‐sup‐0001‐AppendixS1.pdf.

## Data Availability

All lab materials are published on Qubes (Malish et al. 2026). Code associated with this manuscript is available on OSF (Malish 2026). The human subjects assessment data presented in this manuscript cannot be publicly archived, per our Institutional Review Board protocol.
