# Supplementary material for: Teaching Macrosystems Ecology Concepts With a Collaborative, Adaptable Education Module
Source: Ecol Evol. 2026 Jul 1;16(7):e73909. doi: 10.1002/ece3.73909 (PMC13322780; doi:10.1002/ece3.73909)
Supplement: Supplementary file 1 — Appendix S1: ece373909‐sup‐0001‐AppendixS1.pdf. [file ECE3-16-e73909-s001.pdf]

The UNIVERSITY of OKLAHOMA

Consent

Consent to Participate in Research
University of Oklahoma

You are invited to participate in research about hydrology education.

If you agree to participate, you will complete a 5 minute online survey three times (total time of 15 minutes). For this research you will also complete a two part lab activity. Part A takes approximately 60 minutes. Part B will take approximately 30 minutes.

You may experience these risks:
Data collected online or by a device and transmitted electronically: You will be asked to complete an online survey as part of this research. The organization hosting the data collection platform has its own privacy and security policies for keeping your information confidential. There is a risk that the external organization, which is not part of the

research team, may gain access to or retain your data or your IP address which could be used to re-identify you. No assurance can be made as to their use of the data you provide for purposes other than this research.

There are no benefits for participating in this research.

Your participation is voluntary and your responses will be anonymous.

We will not share your data or use it in future research.

Even if you choose to participate now, you may stop participating at any time and for any reason.

If you have questions about this research, please contact: Megan Malish [megan.malish@ou.edu] and Tom Neeson [neeson@ou.edu]

You can also contact the University of Oklahoma – Norman Campus Institutional Review Board at 405-325-8110 or irb@ou.edu with questions, concerns or complaints about your child’s rights as a research participant, or if you don’t want to talk to the researcher.

Please print this document for your records. By providing consent, I am agreeing to participate in this research.

Are you 18 years of age or older?

- ○ Yes
- ○ No

Do you consent to participate in this research?

- ○ Yes
- ○ No

Check

Which statement describes you:

- ○ I have not yet started the Stream Drying and Cross Scale Interactions Lab
- ○ I have completed Part A of the Stream Drying and Cross Scale Interactions Lab. I have not started Part B.
- ○ I have completed Part A and Part B of the Stream Drying and Cross Scale Interactions Lab.

Survey 1

How would you rank your **proficiency** with understanding and interpreting hydrological data?

No proficiency, not able to use this in an assignment	Basic proficiency, able to handle simple applications to an assignment	Intermediate proficiency, able to use this independently for many types of assignments	Advanced proficiency, able to use this independently for nearly all types of assignments	Expert proficiency, able to use this independently for all types of assignments and serve as a role model or coach others
○	○	○	○	○

How would you rank your **confidence** with understanding and interpreting hydrological data?

Not at all confident	Somewhat confident	Moderately confident	Very confident	Completely confident
○	○	○	○	○

What statement best describes your current knowledge of cross-scale interactions?

Not at all familiar, I have never heard of cross-scale interactions	Slightly familiar, I have heard of cross-scale interactions, but cannot elaborate	Somewhat familiar, I could explain a little about cross-scale interactions	Moderately familiar, I could explain quite a bit about cross-scale interactions	Extremely familiar, I could explain and instruct others about cross-scale interactions
○	○	○	○	○

To the best of your ability, describe what 'cross-scale interactions' means in a hydrological context.

The following figure shows two years of data for a watershed. In the figure, what is a possible cause of the reduced percent wet length that occurred between May

and September?

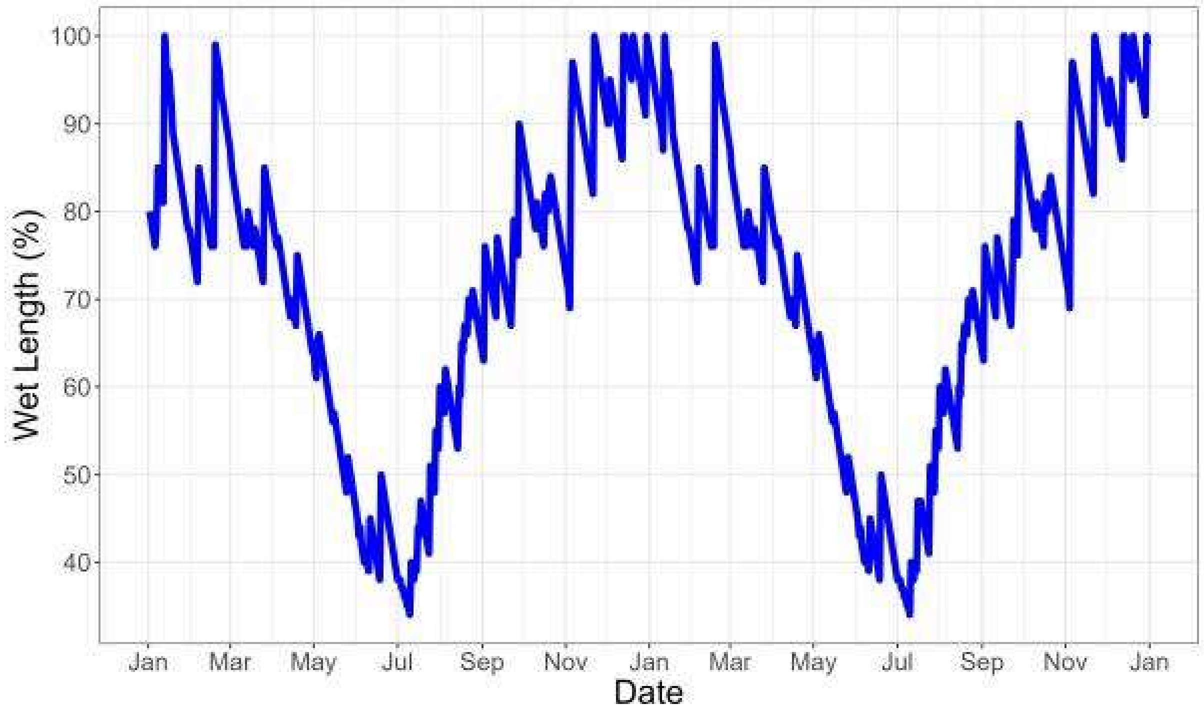

- Climate change occurred from May through September and caused parts of the watershed to dry.
- Low precipitation in May through September reduces the width of the river channel.
- Seasonal patterns of warm temperatures and low precipitation caused parts of the watershed to dry.
- I do not know.

You are modeling stream flow in a watershed with non-perennial reaches over a 5-year period. In the first simulation, you run the model to simulate stream flow for current climate conditions. In the second simulation, you run the model to simulate stream flow for climate conditions expected 50 years from now. How will stream

drying, as measured by percent wet length, differ between the two simulations?

- There is not enough information to know how stream drying will change.
- The watershed will tend to have lower percent wet length measurements in the first simulation.
- The watershed will tend to have lower percent wet length measurements in the second simulation.
- I do not know.

You model stream flow in a watershed for current and future climate conditions and find that stream drying increases under future climate conditions. How would you expect percent wet length and number of dry segments to differ between current and future stream flow simulations?

- Both percent wet length and number of dry segments will be higher in the future.
- Percent wet length will be lower and number of dry segments will be higher in the future.
- Percent wet length will be higher and number of dry segments will be lower in the future.
- I do not know.

Powered by Qualtrics
